# The assessment of stress level, anxiety, depressive symptoms, and defense mechanisms among Polish and English medical students

**DOI:** 10.1186/s12991-020-00274-7

**Published:** 2020-05-03

**Authors:** M. Pawlaczyk, J. Siembida, K. Balaj, A. Rajewska-Rager

**Affiliations:** 1 Department of Adult Psychiatry, Poznan University of Medical Sciences, Poznan, Poland; 2 Psychiatry Students Scientific Group, Poznan University of Medical Sciences, Poznan, Poland

**Keywords:** Medical students, Stress, Anxiety, Defense mechanisms

## Abstract

**Introduction:**

Medical education is proven to be associated with a high degree of psychological stress. Different coping strategies used by students have been investigated on their efficacy. So far, studies on medical students have been limited to a single population.

**Aim of the study:**

Our study aimed to identify differences in the prevalence of depressive symptoms, anxiety, stress levels, and defense mechanisms among two groups of medical students, the Polish and English-speaking divisions.

**Materials and methods:**

The study included two groups of first-year medical students, the Polish and English-speaking divisions, comprising 305 participants (*n* = 204 Polish, *n* = 101 English, men = 127, women = 176). It was divided into two periods: the students received author questionnaires during an exam-free academic period and then completed the same questionnaires during an exam session. The survey contained questions pertaining to demographics and studying habits among participants and included the Defense Style Questionnaire and Depression Anxiety Stress Scales. Data were analyzed using STATISTICA version 12.0, and *p* ≤ 0.05 was considered significant.

**Results:**

Polish medical students presented with significantly increased overall stress levels (*p* = 0.007858) and depressive symptoms (*p* = 0.030420) compared to the English division students. Polish students also presented with more symptoms of stress, depression, and anxiety during the exam period compared to the exam-free period (*p* = 0.000625), which did not apply to the English-speaking students. The English division students reached higher scores in the mature defense mechanisms section than the Polish students (*p* = 0.000001). The use of mature defense mechanisms correlated negatively with the intensity of stress, anxiety, and depressive symptoms in both groups, while immature defense mechanisms promoted higher values of those variables (*p* = 0.000001).

**Conclusions:**

Our study showed significant and multidirectional differences between medical students of the Polish and English divisions attending the same university. Such results could suggest that strategies aimed at reducing depressive symptoms among medical students ought to be adapted towards the needs of a specific population.

## Background

Medical education worldwide is proven to be associated with a high degree of psychological stress [1, 2], a factor that has been found to correlate with depression and anxiety [3]. Students in medical training experience high levels of anxiety more often than the general population of the same age group [2]. Moreover, the prevalence of depressive symptoms in medical students is also significantly higher than in the general population [4]. Consistent with the statements mentioned above, a recent systematic review with meta-analysis determined that the prevalence of depressive symptoms among medical students amounts to 27.2% and suicidal ideation to 11.1% [5]. The knowledge of those facts has led to further investigations on the demographic variables determining stress, anxiety, and depression among medical students [3] and their correlations with academic performance [6]. Saravanan et al. found no significant relationship between gender, year of study or stage of training (preclinical or clinical) with the existence or nonexistence of depression, whereas such a relationship between gender and anxiety was observed—female students presented with anxiety more often than males. The same study indicated stress as a significant predictor of depression. A study led by Erschens et al. showed that perceived stress among medical students at different stages of their medical education was high and differed depending on the year of education, and reached the highest value during the third and ninth semester of medical school [7]. Interestingly, Heinen et al. found no statistically significant differences in medical students’ stress levels regarding gender, employment status, or migration background [8]. Waqas et al. have described significant differences between anxiety levels and depression levels in medical students and the mean percentage of marks obtained in annual examinations. Studies have also determined sources of stress that are specific to the medical curriculum [9]. Consequently, expert recommendations targeting the improvement of medical students’ mental health and well-being began to emerge [10]. For instance, stress management training programs for students of various medical professions have shown to be an effective means in reducing anxiety and stress [11]. Different coping strategies used by students during medical education have also been determined and investigated on their efficacy [9, 12]. Among them were the ego defense mechanisms, defined as unconscious psychological processes that help an individual cope with anxiety resulting from stressful events [6, 13]. Different defense styles contribute to the maturity, health, and adaptiveness of an individual [14]. Recent studies suggest that specific tendencies in the use of the defense mechanisms might be related to some psychiatric diagnoses [15]. Mature mechanisms are associated with problem-solving actions, while immature mechanisms are associated with escape and evasion reactions [16]. A study on medical students proved that a mature defense style was associated with better academic performance, lower anxiety, and lower depression levels while immature and neurotic defense styles were associated with relatively lower academic performance as well as higher anxiety and depression levels [6]. Another study led by Waqas et al. showed a positive correlation between higher scores on mature and neurotic defense style scales and academic achievements [17]. These findings imply that defense mechanisms may have an impact on the outcome of the educational process. So far, to our knowledge, the studies on defense mechanisms of medical students have not compared students from two populations, differing in cultural background and previous environmental influences. There are, however, indications that cultural differences between various medical student populations influence their mental health—specifically depressive symptoms and stress—as well as wellness and quality of life [18]. Our study aimed to identify differences in prevalence, stress levels, anxiety, depression and defense mechanisms among two groups of medical students, the Polish and English-speaking divisions, obtaining medical training under the same environmental circumstances. It also sought for correlations between stress levels, time spent studying and applied defense mechanisms among the students.

## Materials and methods

### Participants

The clinical sample included first-year medical students of Poznan University of Medical Sciences (*n* = 305, male = 127, female = 176). The students were further divided into two subgroups based on the Polish and English-speaking divisions which are independent medical doctor degree programs at the university, (*n* = 204 Polish, *n* = 101 English). Such division resulted from the knowledge of different ethnic, cultural and educational backgrounds of the students—the Polish group being very homogenous in those aspects, the English-speaking group representing various nationalities (including a majority of American, Canadian, Norwegian, Taiwanese, and a minority of others) and a culturally diverse population. A majority of the medical students (66.9%) were from the Polish division, and 33.1% belonged to the English division. All participants completed questionnaires during two different time periods, first an exam-free academic period and then an academic exam session. Only first-year medical students were included in both study groups in order to ascertain the participants were possibly the most study-naive and least unified regarding coping mechanisms. As the study was divided into two different time periods, the first sample size included 104 Polish students and 51 English students. Fewer returning participants in the second part of the study contributed to one less student in the English division (50) and four fewer students from the Polish division (100). The combined sample size was equal to 305 participants (*n* = 305) as mentioned above. Demographic data obtained from questionnaires revealed a total of 176 female participants (57.7%) and 127 male participants (41.6%). The remaining 2 participants (0.7%) failed to declare their sex. Age varied widely among first-year medical students, from 19 years of age to 49 years of age. The majority of students, however, were 20 years of age (54.8%) which closely resembled the average age of all participants, 21.7. Demographic data are summarized in Table 1.

**Table 1:** Demographic data

	Number	Percentage
Division		
Polish	204	66.9
English	101	33.1
Sex		
Male	127	41.6
Female	176	57.7
Undeclared	2	0.7
Age		
19	8	2.6
20	167	54.8
21	36	11.8
22	14	4.6
23	13	4.3
24	22	7.2
25	8	2.6
26	9	3
27	11	3.6
28	2	0.7
29	3	1
31	1	0.3
36	2	0.7
49	2	0.7
Undeclared	7	2.3

All participants received detailed verbal explanations regarding the aim of the study. Subjects provided verbal informed consent before completing questionnaires designed to collect data from medical students. Questionnaires were distributed during class time, and the surveys were also available online for students to complete on their own time.

## Method

The study was divided into two important time periods and carried out between April and June 2016. First, medical students received questionnaires during an exam-free academic period (early spring). Second, the students received the same questionnaires during a heavy exam period at the end of the semester (late June). The survey consisted of a series of questions about demographics and significant studying habits among participants. Time spent studying daily, subjective stress levels, adjuvants during studying, and possible symptoms of mood disorders were included in the survey. It also consisted of the Defense Style Questionnaire (DSQ-40) and the Depression Anxiety Stress Scales (DASS).

The Defense Style Questionnaire (DSQ-40) consisted of 40 items designed to identify mature, neurotic, and immature defense mechanisms. The shortened version of the original DSQ-88 proved to be reliable and efficient while permitting enough questions to allow discrimination of defenses [19]. Thus, the participating subjects were asked to use a 9-point severity scale (1–9) to declare if the item applied to them. The higher the number circled, the more that certain item was identifiable with the participant. Scores were summed and analyzed for statistical significance. The Depression Anxiety Stress Scales (DASS) consisted of 42 items that identified three specific emotional states of depression, anxiety, and stress [20]. The participating subjects declared on a 4-point (0–3) severity scale how likely the statement applied to them within the past week. A higher score indicated that the statement “applied to me very much, or most of the time”. A score of 0 indicated that the statement did not apply to the participant at all. The Depression scale assessed symptoms such as dysphoria, devaluation of life, and anhedonia. The Anxiety scale assessed symptoms such as autonomic arousal and situational anxiety. The stress scale assessed difficulty relaxing, nervous arousal, and being easily agitated and impatient. Scores for depression, anxiety and stress were summed for statistical analysis.

### Statistical analysis

The data were compiled in Microsoft Excel and analyzed with appropriate statistical tests using Microsoft Excel and STATISTICA 12.0 PL (StatSoft Polska, Kraków, Poland). Analyzed data were presented as a mean and a standard deviation, or as a median and an interquartile range (upper and lower quartiles), or as absolute numbers or percentages, as appropriate. Normality of the distribution was tested with the Shapiro–Wilk’s test, and the equality of variances was checked with the Levene’s test. Data that did not follow a Gaussian distribution were analyzed with Mann–Whitney. The relationship between variables was analyzed with the Spearman’s rank correlation coefficient. In all analyses, a *p*-value of < 0.05 was considered to be statistically significant.

## Results

### Time spent studying, subjective stress level, depressive symptoms

**Fig. 1:**
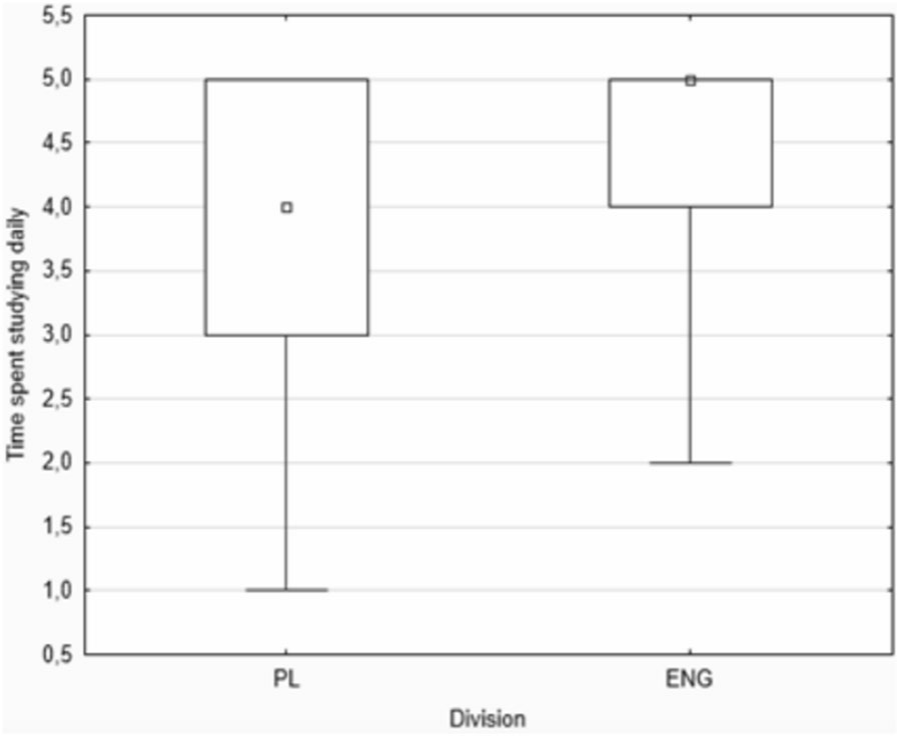
Time spent studying daily

The English division students generally spent more time studying daily than the Polish students, with the median being 5 h and 4 h, respectively (Fig. 1). Interestingly, the majority of respondents (52.1%) included from both groups admitted to studying more than 5 h daily.

Moreover, the Polish group showed differences in time spent studying before (median = 3 h) and during (median = 5 h) a heavy exam period, while the English group did not show such a difference. Both student groups reported similar levels of subjectively perceived stress, the median being seven on the scale 1–10. However, again, the Polish division reported differences in the levels of subjectively perceived stress before and during the exam period, with the median being 5 and 8, respectively, whereas no such differences were observed in the English division students.

**Fig. 2:**
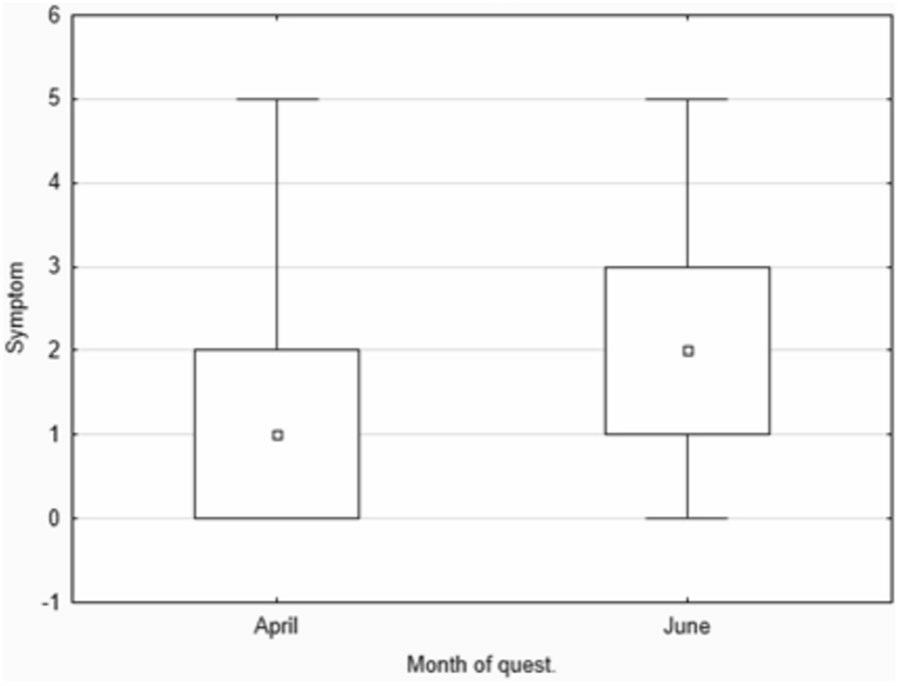
Depressive symptoms (non-exam period: April vs. exam-heavy period: June)

The studied groups did not differ significantly in the number of subjectively experienced depressive symptoms at any time. Again, when analyzed selectively, the Polish group presented with significantly more depressive symptoms during the exam period compared to the non-exam period (*p* = 0.006952), while the English group did not present noticeable differences there. The differences in reported depressive symptoms among Polish students depending on the academic period are presented in Fig. 2.

### The depression anxiety stress scales

The students belonging to the Polish division presented with significantly increased overall stress levels (*p* = 0.007858) and depressive symptoms (*p* = 0.030420) compared to the English medical students. The Polish population also experienced significantly higher levels of stress (*p* = 0.000121), depressive symptoms (*p* = 0.000734) and anxiety (*p* = 0.015417) during a heavy exam period compared to exam-free period, which failed to apply to the English division. Regardless of the analyzed group, female students presented with higher scores on the DAS Scales. Female students of the Polish division displayed higher levels of anxiety (*p* = 0.004682) and stress (*p* = 0.000598) than male students. Similarly, statistically significant differences in levels of depression (*p* = 0.007282), anxiety (*p* = 0.009599), and stress (*p* = 0.007441) were seen between English division students when assessing differences in gender. Interestingly, a positive correlation between objective stress (the S factor on the DASS scale) levels and time spent studying daily was only seen among the Polish group. Specific data pertaining to the findings mentioned above are presented in Tables 2, 3, and 4.

**Table 2:** Depression, anxiety, stress n PL vs. ENG (MP2-5.08.16)

	Division	*N*	Average	Median	Minimum	Maximum	Lower quartile	Upper quartile	SD
d	PL	204	10.46	8.00	0.00	40.00	3.00	16.00	8.60
a	PL	204	10.05	8.00	0.00	28.00	4.00	15.00	7.09
s	PL	204	15.89	15.00	0.00	38.00	8.00	23.00	9.87
d	ENG	101	8.93	6.00	0.00	42.00	2.00	13.00	9.24
a	ENG	101	9.63	7.00	0.00	34.00	2.00	16.00	9.34
s	ENG	101	12.85	11.00	0.00	42.00	5.00	20.00	9.97

*d* depression,* a* anxiety,* s* stress,* n* number,* SD* Standard deviation

**Table 3:** Depression, anxiety, stress in males vs. females (PL Division)

PL	Sex	*N*	Average	Median	Minimum	Maximum	Lower quartile	Upper quartile	SD
d	m	75	9.69	7.00	0.00	32.00	3.00	15.00	8.36
a	m	75	8.28	6.00	0.00	26.00	3.00	13.00	6.64
s	m	75	13.04	11.00	0.00	38.00	4.00	20.00	9.97
d	f	129	10.90	8.00	0.00	40.00	4.00	16.00	8.74
a	f	129	11.09	11.00	0.00	28.00	5.00	15.00	7.16
s	f	129	17.54	17.00	0.00	37.00	10.00	25.00	9.46

**Table 4:** Depression, anxiety, stress in males vs. females (ENG Division)

ENG	Sex	*N*	Average	Median	Minimum	Maximum	Lower quartile	Upper quartile	SD
d	m	52	7.,42	3.00	0.00	34.00	1.00	10.50	9.49
a	m	52	7.38	3.00	0.00	31.00	1.00	11.00	8.42
s	m	52	10.25	7.50	0.00	32.00	3.00	15.00	9.20
d	f	47	10.38	8.00	0.00	42.00	3.00	14.00	8.90
a	f	47	12.04	10.00	0.00	34.00	3.00	20.00	9.77
s	f	47	15.53	15.00	0.00	42.00	7.00	22.00	10.16

### The Defense Style Questionnaire

The English division students reached significantly higher scores (*p* = 0.000001) in the mature defense mechanisms section (mean 5.66; SD = 1.28) than the Polish students (mean 4.90; SD = 1.23). Mature mechanisms mentioned included suppression, sublimation, humor, and anticipation. The difference was noticeable both before and during the heavy exam period.

The comparison of significant differences in the DSQ results between both studied populations is presented in Table 5.

**Table 5:** Comparison of the DSQ Results Between Both Studied Populations

	Average- PL	Average- ENG	*t*	Df	*p*	*n* = PL	*n* = ENG	Standard deviation = PL	Standard deviation = ENG
M	4.901348	5.659653	− 4.99234	303	0.000001	204	101	1.230824	1.283442
I	3.648849	4.216172	− 4.47223	303	0.000011	204	101	0.989319	1.143243

*M* mature defense mechanisms, *I* Immature Defense Mechanisms

*t*-Test; Group: Division (MP2-5.08.16) Group 1: PL Group 2: ENG

Interestingly, there was no statistical difference in both study groups between any applied defense mechanism during the exam period. Both the English and Polish division students tended to apply mature, immature, and neurotic defense mechanisms with similar intensity regardless of the academic period. Neurotic defense mechanisms included undoing, pseudo-altruism, idealization, and reaction formation and immature defense mechanisms included projection, passive aggression, acting out, isolation, devaluation, autistic fantasy, denial, displacement, dissociation, splitting, rationalization, and somatization. Statistically significant differences between males and females regarding neurotic and mature defenses were noted. Among the Polish group of medical students, females obtained a higher score in the neurotic defense mechanisms (mean 4.13) compared to males (mean 3.5). Also, males in both groups applied mature defense mechanisms (mean = 5.5) more often than females (mean = 5.0).

Thus, the use of mature defense mechanisms correlated negatively, but weakly, with the intensity of stress (*R*—Spearman = − 0.126763), anxiety (*R*—Spearman = − 0.158005) and depressive symptoms *R*—Spearman = − 0.125758) in both groups. Immature defense mechanisms, in contrast, promoted higher values of the above-mentioned variables: stress (*R*—Spearman = 0.282345), anxiety (*R*—Spearman = 0.291349) and the strongest correlation with depressive symptoms (*R*—Spearman = 0.341762).

### The use of adjuvants

In addition to DSQ and DASS scales, study subjects also declared their use of adjuvants aimed to improve learning efficacy. Both study groups declared similar usage of adjuvants. The most common reported adjuvant was coffee, with 72.13% of students using it, followed by caffeine (34.09%) and dietary supplements (24.26%).

Subjectively experienced stress levels were influenced differently among the Polish and English division students. For instance, the use of any adjuvant correlated with a higher level of subjectively perceived stress in the Polish group (*R*—Spearman 0.252437, *p* = 0.000270), while no such correlation existed in the English group. A positive correlation existed between higher subjective stress levels and the use of caffeine when considering all of the subjects (*p* = 0.002112).

The use of adjuvants also correlated positively, but weakly, with the intensity of applied neurotic and immature defense mechanisms (*R*—Spearman, respectively, 0.14 and 0.15) in both study groups, meaning that the students presenting less effective defense mechanisms tended to consume more adjuvants. This tendency, while weak, is consistent with a study showing that internet addiction among medical students correlates positively with less effective defense mechanisms [21]. 
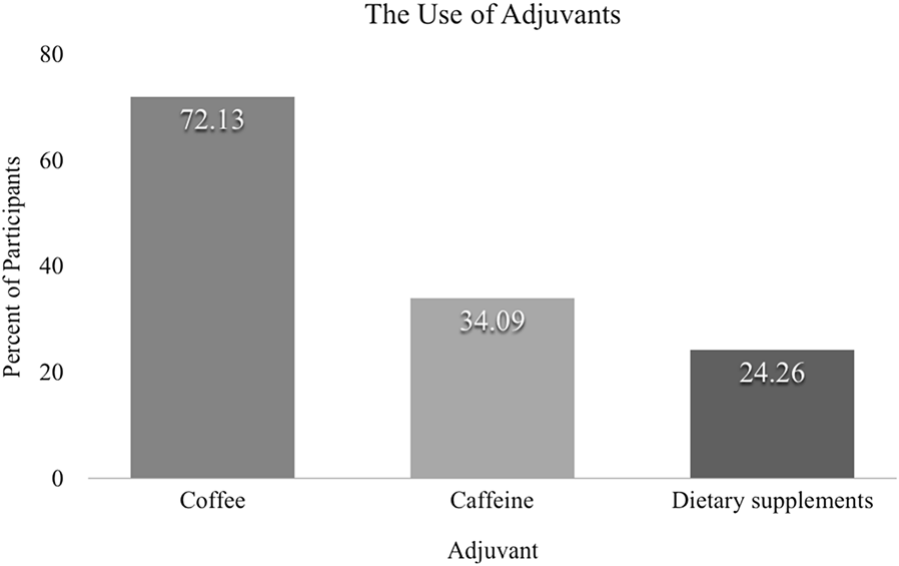


## Discussion

There is growing evidence that the process of obtaining medical education puts a great strain on students, resulting in high levels of stress, as well as a significant prevalence of depressive symptoms and suicidal ideation among them [1–3, 5]. In a recently published meta-analysis, no significant differences in prevalence estimates of depression and depressive symptoms in medical students were found when comparing studies performed in and outside the United States [5]. In the context of those results, our study combined two distinct aspects of the previous research—it sought to compare students with different ethnic and cultural backgrounds attaining medical education under the same environmental conditions. Given that the subjects were recruited early on during their academic experience, the observed differences between the two groups are substantive regarding the influence of culture and background, rather than solely considering environmental factors when comparing the two groups.

As a foremost finding, our study has shown significant differences in depressive symptoms and stress levels between the Polish and English division students. More importantly, the Polish group displayed differences in the intensity of experienced symptoms depending on the academic period (exam versus exam-free period), while the English-speaking group did not. This could suggest that even under homogeneous circumstances of obtaining an education, being the same university, different populations of students would still vary in presented reactions to stressors. Hofmann et al. stated that a person’s cultural background influences the experience and expression of emotions [22], consistent with one of the many possible factors for the observed differences. The results of our study imply that factors such as cultural conditioning and previously acquired psychological resources play an important role in emotional regulation and reaction to stress. The above-mentioned factors constitute, among numerous others, resilience resources and thus influence an individual’s response to stress [23]. Together with socioeconomic status and past instructive experiences with adversity, these factors are likely to differ strongly between the two studied populations, contributing to significant differences in stress levels among the students. Other significant factors influencing the reactions of the studied populations to education**-**related stressors are varying experiences and exposures to academic training among the two groups. The vast majority of Polish division students begin medical studies directly after graduating high school, whereas many of the English division students graduate from various colleges or complete other forms of secondary training before applying to medical school. This difference was noted to be important in a study comparing medical education in Germany (which is the same as in Poland) and the United States [24]. That additional experience of academic studying may benefit the English division students by allowing more time and opportunities to adapt, resulting in more effective coping strategies during medical education. Slight differences in academic exam periods between the English and Polish division likely influenced the outcome of results as well. Another factor possibly influencing varying reactions to stressors among both groups was age (median being 20 in the Polish group and 24 in the English group)—as it increases the personal experience of the subjects and thus plays a role in developing different coping strategies. As reported in numerous studies, age significantly predicts the use of problem-focused engagement [25]. Specifically, older students are more likely to use problem-focused strategies. The results of our study were consistent with this tendency. Although the association between age and emotional response to stress is neither direct nor unequivocally determined, there are some indications that older age fosters less negative responses in reaction to stressors [26]. Finally, we hypothesize that the English division students constitute a more resilient and resourceful population than Polish division students, given the fact that they were willing to undertake higher education in a foreign country, without the support of their social network and possibly by anticipating experiencing some levels of acculturative stress, apart from the education-related stressors.

Regardless of the studied group, subjects striving for academic success with increased stress and anxiety were noted to have thoughts of resignation more frequently. This result suggests that strategies targeting stress reduction in students could reduce depressive symptoms or even prevent their onset in some cases.

In both studied populations, female subjects tended to reach higher levels of stress and anxiety than male students. These results are consistent with earlier research on medical students [27], where Qamar et al. hypothesized that specific psychosocial profiles could explain higher stress in female students. Our results suggest that regardless of their environmental background, female students are prone to react more sensitively to stressors present in the academic educational system. Further research on the influence of gender on coping with stress among medical students could determine if other factors such as hormonal differences or environmental pressure contribute to the discrepancies as well.

Concerning caffeine use in our study subjects, the results are consistent with a study on caffeine consumption and self-assessed stress, anxiety, and depression. The study reports that caffeine consumption may be associated with the factors mentioned above [28]. Study subjects using caffeine (including coffee) as an adjuvant were noted to use neurotic and immature defense mechanisms more often than mature defense mechanisms. They also reported feelings of resignation more often than the subjects who were not using stimulants (49.84% vs. 7.87%). Thus, our study obtained evidence of increased stress with caffeine use in medical students of the English and Polish division.

When analyzing the ego defense mechanisms, we found that higher values of the DAS Scale variables were present among students applying immature and neurotic defense mechanisms more often. On the contrary, students applying mature defense mechanisms presented overall as less stressed, anxious, and depressed. These results are consistent with the concept of psychological defense mechanisms and previously conducted research [6, 16]. An interesting finding was that the English division students presented with more mature defense mechanisms than the Polish students. This result, again, might partially be a consequence of a statistically older English-speaking group. Mature defense mechanisms are known to be positively correlated with better academic performance [6]. Based on our results, males in both study groups applied mature defense mechanisms, which may be used to predict higher academic performance. Females in our study groups portrayed a higher number of neurotic defense mechanisms which are correlated with poor academic performance [6].

The results of our study showed that there were significant and multidirectional differences between medical students of the Polish and English division, even though both groups attended the same university. These findings add new data to the research determining the causes of stress and coping strategies among medical students of different nationalities [29–31]. They also implicate that strategies aimed at reducing depressive symptoms among medical students ought to be adapted towards the needs of a specific population. In our study groups, Polish students would benefit more from stress reduction during a heavy exam period than the English division students, who did not show a significant disparity in depressive symptoms before and during exam sessions.

The limitations of our study include its cross-sectional nature and, similarly to other questionnaire based research, the reliability of student responses. Also, the correlations observed in our study may not be applicable to other universities, as they constitute a different environmental background.

## Data Availability

The datasets during and/or analyzed during the current study are available from the corresponding author on reasonable request.
